# Low- to Middle-Income Nations: Resource Rich in People but Resource Poor in Reliable Utility (electrical grid) Infrastructure for Effective Delivery of High-Impact Supportive Care Modalities—Some Thoughts on the Radiotherapy Perspective

**DOI:** 10.1200/JGO.18.00082

**Published:** 2018-07-11

**Authors:** George A. Dawson

**Affiliations:** **George A. Dawson**, James J. Peters VA Medical Center, Bronx, NY.

## TO THE EDITOR:

The article by Grover et al^1^ highlights the increasing sophistication of health care providers in delivering different aspects of palliative care in resource-challenged nations. These nations are typically resource rich in people and natural resources but resource poor in regard to infrastructure development that, in this case, relates to the delivery of supportive care, especially that for cancer care.

In my opinion, the authors have created a workable model for the development of supportive care delivery. However, they make no mention of the role of radiotherapy in their model. Radiotherapy is used in 60% of patients with cancer at some point during their illness.^2^ If palliative external beam radiotherapy is used judiciously and according to established clinical guidelines, it can offer verifiable relief of pain with just a single fraction of radiation.^3^

India, the second most populous nation in the world, is still listed among resource-poor nations. For example, India lacks the electrical infrastructure to effectively and reliably operate modern high-demand linear accelerators. This, combined with the increasing financial demands of growing populations and increasing numbers of cancer cases, challenges the health care system in this nation because the country cannot provide a sustainable (electrical) infrastructure and the financial resources needed to support high-tech radiotherapy machines. Currently, India has only one third of the radiotherapy machines needed to treat their patients with cancer.^4^

Nigeria, with its population of 200 million is similar to India in terms of resource-constrained infrastructure but is less populous. Accessibility to radiotherapy delivery systems is severely limited. Nigeria also lacks the necessary infrastructure (electrical and financial) to provide this vital aspect of supportive cancer care, although it does have eight radiotherapy delivery machines located in seven different hospitals that can be used to treat patients.^5^

Kumar and Bhasker^6^ have argued that rather than focusing on acquiring high-tech linear accelerators with their inherent technical demands and financial cost, developing a more modernized cobalt 60 treatment delivery system may be less expensive and more durable for nations like India. This is an idea worth considering in view of the current upheavals because of war and the lack of life-sustaining jobs in these resource-poor nations.
